# Clinical Cases

**Published:** 1885-11

**Authors:** E. W. Berridge

**Affiliations:** London


### CLINICAL BUREAU.

### CLINICAL CASES.

### E. W. Berridge, M. D., London.

(1.) *Anantherum muricatum*.—1884, February 25th.—Miss Kate A., æt. twenty-eight.—For five days feeling of a ball in the head; worse on moving head at night, when lying on the right side, and when stooping. The pains seemed to have been caused by catching cold five days ago. There was also throbbing in vertex and occiput; gave her a dose of *Ananth. muricatum*$^{cm}$ (Swan) at nine P. M., and told her to repeat the dose every four hours till relieved; supplied her with medicine for four days.

March 10th.—Wrote to say that the pains in head were much better the next morning, so that on the following day the ball pains went and did not return. By the twenty-eighth all the pains in head had quite left her.

1885, January 30th.—Her sister, whom I had cured of a fissure in rectum, reports that patient had remained perfectly well.

*Anantherum* is one of Houat's provings, concerning which I will have something to say later. Houat's symptoms have been omitted in every repertory that I have examined for them, even in Allen's *Index*, though incorporated in his *Encyclopædia*. Fortunately I found them in my MS. Repertory of Head Symptoms. It is noteworthy that Dr. Richard Hughes, who is now posing before the profession as an improver of HAHNEMANN'S Homœopathy and a purifyer (?) of the materia medica, stigmatizes these provings as "actual lies." Can the new *Cyclopædia of Drug Pathogenesy*, and the new *Materia Medica, Physiological and Applied*, edited by such an unscrupulous slanderer, be anything but a miserable caricature?

(2.) *Oxygen.*—1885, January 17th.—Cough excited by tickling in throat, and causing soreness of chest, occurring between two and three A. M., and better when lying on the back; is promptly cured by one dose of *Oxygen*$^{cm}$ (Swan), thus verifying Swan's proving of this remedy published in *The Organon.*

